# Relationships between serum-induced AhR bioactivity or mitochondrial inhibition and circulating polychlorinated biphenyls (PCBs)

**DOI:** 10.1038/s41598-017-09774-1

**Published:** 2017-08-24

**Authors:** Wook Ha Park, Sora Kang, Hong Kyu Lee, Samira Salihovic, Bert van Bavel, P. Monica Lind, Youngmi Kim Pak, Lars Lind

**Affiliations:** 1 Department of Physiology, College of Medicine, Kyung Hee University, Seoul, 02447 Korea; 2 Department of Internal Medicine, College of Medicine, Eulji University, Seoul, 01830 Korea; 3 MTM Research Centre, School of Science and Technology, Örebro University, Örebro, SE-701 82 Sweden; 4 Occupational and Environmental Medicine, Uppsala University, Uppsala, SE-751 05 Sweden; 5 Department of Medicine, Cardiovascular Epidemiology, Uppsala University, SE-751 05 Uppsala, Sweden

## Abstract

Metabolic syndrome and mitochondrial dysfunction have been linked to elevated serum levels of persistent organic pollutants (POPs). However, it is not clear which specific POPs contribute to aryl hydrocarbon receptor (AhR)-dependent bioactivity or inhibit mitochondrial function in human subjects. Here, we measured the cumulative bioactivity of AhR ligand mixture (AhR bioactivity) and the effects on mitochondrial function (ATP concentration) in recombinant Hepa1c1c7 cells incubated with raw serum samples obtained from 911 elderly subjects in the Prospective Investigation of the Vasculature in Uppsala Seniors (PIVUS) cohort. Plasma concentrations of 30 POPs and plastic chemicals have previously been determined in the same PIVUS subjects. Linear regression analysis demonstrated that total toxic equivalence (TEQ) values and polychlorinated biphenyls (PCBs) were significantly correlated with AhR bioactivity (positively) and ATP concentration (negatively). Serum AhR bioactivities were positively associated with some PCBs, regardless of their dioxin-like properties, but only dioxin-like PCBs stimulated AhR bioactivity. By contrast, PCBs mediated a reduction in ATP content independently of their dioxin-like properties. This study suggests that AhR bioactivity and ATP concentrations in serum-treated cells may be valuable surrogate biomarkers of POP exposure and could be useful for the estimation of the effects of POPs on human health.

## Introduction

Persistent organic pollutants (POPs) include a wide range of lipophilic chemicals that persist in the environment and accumulate in human adipose tissue, having potential risk for human health^1, 2^. Although the use of some POPs was prohibited in most countries following the 2001 Stockholm Convention on POPs, considerable quantities of polychlorinated biphenyls (PCBs), dioxins and organochlorine pesticides (OCPs) are still found in the environment, and also in some people. Epidemiological studies revealed that high serum concentrations of various circulating POPs are strongly associated with obesity^3^, diabetes^4, 5^, neurological disorders^6^, inflammatory diseases^7^, hypertension and dyslipidaemia^8^. However, it is difficult to establish cause-effect relationships between POPs and human diseases because the composition and amounts of the POPs present are enormously diverse, vary greatly over time and can exert additive or synergistic effects in combination^9^. The levels of POP present were determined by high-resolution gas chromatography coupled to high-resolution mass spectrometry (HRGC/HRMS), a conventional chemical method for POP quantitation, in all these epidemiological studies. Studies of human subjects aimed at verifying cause-effect relationships have been limited to date because the HRGC/HRMS method requires a large amount of serum or plasma for organic solvent extraction and detects only the designated chemicals among several hundred candidates. Therefore, it would be useful to develop a good serum biomarker that could indicate the potential bioactivity or health effects of serum POPs.

We previously reported two different cell-based assays that quantify POP levels and mitochondrial inhibition in small volumes (10 μl) of heat-inactivated raw human serum: a cell-based aryl hydrocarbon receptor (AhR) ligand activity assay (CALA) and an ATP assay^10^. AhR is a cytosolic nuclear receptor that binds many xenobiotic ligands, including dioxins^11–13^. Agonist ligand-bound AhR forms a heterodimer with the AhR nuclear translocator (ARNT, also known as hypoxia-inducible factor 1β, HIF1β) and activates the transcription of multiple genes, which enhance the metabolism and clearance of AhR ligands by binding dioxin-response elements (DREs) in target gene promoters. Thus, the AhR-DRE-driven reporter activity in cells incubated with raw serum, AhR bioactivity, reflects the cumulative bioactivity of an AhR ligand mixture in a serum sample.

In parallel, measurement of the ATP concentration in cells incubated with serum indicates the effects of the serum sample on mitochondrial function. Using the CALA and ATP assays, we previously demonstrated that serum AhR bioactivity is elevated but ATP levels are reduced in subjects with diabetes and impaired glucose tolerance^10^. Independent of this, serum AhR bioactivity (n = 25) was linearly correlated with the total toxic equivalences (TEQs), which were calculated from the concentrations (pg/g lipid) of 17 HRGC/HRMS-determined dioxin congeners^10^, multiplied by their toxic equivalency factor (TEF) values^14^. However, it is not clear whether other POPs, for example, PCBs, may contribute to TEQs and/or AhR bioactivity in combination.

In addition, it has not been determined which individual serum POPs are responsible for the serum AhR bioactivity and/or ATP levels. The ideal approach to the identification of the most important POPs would be to measure AhR bioactivity and ATP concentration in cells treated with human serum samples for which individual POP levels have already been measured. The Prospective Investigation of the Vasculature in Uppsala Seniors (PIVUS)^1^ is a population-based cohort of elderly men and women living in Uppsala, Sweden, which was initiated in 2001. To investigate the predictive power of blood levels of environmental contaminants for human health, a large set of environmental contaminants, including 16 PCBs, three OCPs (p,p’-dichlorodiphenyl-dichloroethylene (DDE), trans-nonachlor and hexachlorobenzene (HCB)), octachlorodibenzo-p-dioxin (OCDD), brominated flame retardant (BDE), bisphenol A (BPA) and perfluoroalkyl substances (PFASs), have been quantified in plasma^15, 16^.

In the present study, the AhR bioactivity and ATP concentration were measured in cells after treatment with PIVUS serum samples (n = 911), and the relationships between AhR bioactivity or ATP concentration and calculated TEQ or individual POPs were analysed to identify the most important POPs. In addition, serum-induced AhR bioactivity and ATP concentration were evaluated for use as surrogate biomarkers of POP exposure.

## Results

### Characteristics of the study population

Among the 1,016 participants in the PIVUS cohort, valid measurements of serum-induced AhR bioactivity and ATP concentration existed for 911 subjects. The mean values for all participants were a 2.18 ± 0.24-fold induction for AhR bioactivity and an 80.5% ± 8.3% (% Control) for ATP concentration, compared with a charcoal-stripped human serum (CS-HS)-treated control. A wide range of concentrations of the studied POPs was reported in the PIVUS cohort^15^. Detection rates for all POPs were relatively high, demonstrating long-term accumulation and persistence of the POPs studied. On average, at least 20 compounds were detectable in each participant. Median values and interquartile ranges (IQR, 25^th^–75^th^ percentile) for the non-lipid-adjusted concentrations (pg/ml) of POPs are summarised in Table 1. Among the detected PCBs, three “non-dioxin-like PCBs” (non-DLPs), PCBs 153, 180 and 138, were detected at the highest median concentrations, while those with the lowest median concentrations were PCBs 189, 206 and 209.

**Table 1:** Median and interquartile range (IQR, 25^th^ and 75^th^ percentile) values for the studied POP variables. The limit of detection (LOD) is given in the brackets.

Variable	Class	N	Median (IQR)
AhR bioactivity (FI)		911	2.1 (2, 2.3)
ATP (% Control)		911	80.3 (75, 86.1)
TEQ_total_^a^	total	980	6.1 (3.8, 9.6)
TEQ_planar_^a^	coplanar & non-ortho	986	6.0 (3.7, 9.5)
TEQ_ortho_^a^	mono-ortho	991	0.18 (0.13, .22)
PCB74^a^	Non-DLP	992	91.4 (63.9, 128.1) [LOD 8.5]
PCB99^a^	Non-DLP	992	90.8 (62.4, 131.9) [LOD 10.0]
PCB138^a^	Non-DLP	992	819.3 (619.2, 1115.8) [LOD 108.7]
PCB153^a^	Non-DLP	992	1427.6 (1114.4, 1847.9) [LOD 117.7]
PCB170^a^	Non-DLP	992	497.5 (385.7, 633) [LOD 36.9]
PCB180^a^	Non-DLP	992	1165.4 (917.8, 1487.8) [LOD 65.3]
PCB194^a^	Non-DLP	992	119.4 (87.6, 158.9) [LOD 4.2]
PCB206^a^	Non-DLP	992	26.8 (20.8, 35.2) [LOD 0.8]
PCB209 ^a^	Non-DLP	992	26.2 (19.6, 34.7) [LOD 1.4]
PCB105^a^	DLP, ortho	992	32 (21, 46.8) [LOD 5.9]
PCB118^a^	DLP, ortho	991	200.6 (136.4, 281) [LOD 25.3]
PCB156^a^	DLP, ortho	992	154.3 (118.7, 197.6) [LOD 10.8]
PCB157^a^	DLP, ortho	992	28 (21.4, 37) [LOD 1.5]
PCB189^a^	DLP, ortho	992	19.2 (14.6, 25.8) [LOD 1.7]
PCB126^a^	DLP, planar	986	40.4 (21.6, 72) [LOD 8.0]
PCB169^a^	DLP, planar	986	171.4 (130.6, 219.8) [LOD 17.5]
OCDD^a^	dioxin	987	2.6 (1.4, 4.2) [LOD 1.4]
BDE47^a^	flame retardant	992	12.6 (9, 19.5) [LOD 9.2]

^a^Serum POPs levels and TEQ are given in pg/ml. Lipid-normalized data are given in the reference^15^ for comparison with other studies.

FI, fold induction; PCB, polychlorinated biphenyl; OCDD, octachlorinated dibenzo-p-dioxin; BDE, brominated diphenyl ether; TEQ, total toxicity equivalent; TEQ_planar_, TEQ for dioxin-like coplanar, non-ortho-substituted PCBs; TEQ_ortho_, TEQ for dioxin-like mono-ortho-substituted PCBs; DLP, dioxin-like PCBs. DLP, dioxin-like PCB, non-DLP, Non-dioxin-like PCB.

### Relationships between PCBs and AhR bioactivity

To determine whether individual POPs were directly correlated with serum AhR bioactivity, we performed a linear regression analysis of AhR bioactivity with the plasma concentrations of 30 POPs, comprising 16 PCBs, OCDD, BDE47, BPA, three OCPs and eight PFASs. As shown in Table 2, the log_e_-transformed TEQ_total_ (adjusted for sex, serum cholesterol and serum triglycerides) was significantly correlated with AhR bioactivity (*P* = 0.012). When the TEQ values were grouped into TEQ_ortho_ for mono-ortho-substituted PCBs and TEQ_planar_ for coplanar and non-ortho PCBs, both TEQ_ortho_ (*P* = 0.0046) and TEQ_planar_ (*P* = 0.013) were also significantly correlated with AhR bioactivity.

**Table 2:** Relationship of serum AhR bioactivity with TEQ or plasma levels of individual POPs (ln-TEQ, ln-POPs, pg/mL) are represented by regression coefficients (β) with corresponding 95% CIs and *P* values (n = 911). Linear regression models were adjusted for sex, serum cholesterol, and serum triglycerides.

Variable	β coefficient (95% CI)	*P* value
TEQ_total_	0.033 (0.0075, 0.059)	0.012*
TEQ_planar_	0.032 (0.0069, 0.058)	0.013*
TEQ_ortho_	0.065 (0.020, 0.11)	0.0046*
PCB74	0.038 (0.0056, 0.071)	0.022*
PCB99	0.031 (0.0019, 0.060)	0.037*
PCB138	0.046 (0.009, 0.084)	0.015*
PCB153	0.053 (0.012, 0.095)	0.012*
PCB170	0.065 (0.019, 0.11)	0.0064*
PCB180	0.057 (0.011, 0.10)	0.016*
PCB194	0.012 (−0.0093, 0.034)	0.27
PCB206	0.040 (−0.0028, 0.083)	0.068
PCB209	0.018 (−0.021, 0.056)	0.37
PCB105	0.025 (−0.0027, 0.053)	0.077
PCB118	0.035 (00032, 0.066)	0.031*
PCB156	0.055 (0.013, 0.097)	0.011*
PCB157	0.065 (0.026, 0.10)	0.0012*
PCB189	0.032 (0.0069, 0.057)	0.013*
PCB126	0.020 (0.0015, 0.038)	0.034*
PCB169	0.029 (−0.0069, 0.065)	0.11
OCDD	−0.0094 (−0.037, 0.018)	0.50
BDE47	0.032 (0.0079, 0.056)	0.0093*

^*^Indicates significant *P* value. All variables were natural log-transformed.

CI, confidence interval; TEQ, toxic equivalence; PCB, polychlorinated biphenyl; TEQ_planar_: TEQ for dioxin-like coplanar, non-ortho-substituted PCBs; TEQ_ortho_: TEQ for dioxin-like mono-ortho-substituted PCBs; OCDD, octachlorinated dibenzo-p-dioxin; BDE, brominated diphenyl ether.

When 16 PCBs were individually analysed, 11 PCBs correlated with AhR bioactivity (*P* < 0.05), with PCB157 showing the lowest *P*-value (*P* = 0.0012) (Table 2). Interestingly, both DLPs (PCBs 118, 156, 157, 189 and 126) and non-DLPs (PCBs 74, 99, 138, 153, 170 and 180) were positively correlated with AhR bioactivity. OCDD, the only dioxin present at detectable levels in most subjects, was not significantly correlated with AhR bioactivity (*P* = 0.50), while the flame retardant BDE47 was significantly correlated (*P* = 0.0093). However, BPA, three OCPs and PFAS^16, 17^ showed no significant correlations with AhR bioactivity (Supplementary Fig. 1).

The present results were obtained using wet-mass concentrations of the POPs in the statistical model, after adjustment for sex, serum cholesterol and serum triglycerides. However, similar results were obtained when lipid-normalised concentrations of the POPs were used in these analyses. No interactions between sex and TEQ were found with respect to AhR bioactivity (*P* = 0.69 for the interaction term). Further adjustment for a history of myocardial infarction, stroke, heart failure, diabetes, and medication for hypertension, diabetes or hyperlipidaemia only had a marginal effect on the correlations between AhR bioactivity and TEQ or individual POPs.

### Relationships between PCBs and ATP concentration

Next, we evaluated the associations of serum effects on cellular ATP concentration with TEQ or individual POP concentration (adjusted for sex and serum lipids) in linear regression models. TEQ_total_ values were inversely correlated with ATP concentration (*P* = 0.0004, Table 3), demonstrating negative effects on mitochondrial function. When the PCBs were analysed separately, both planar DLPs (PCBs 126 and 169), and non-DLPs (PCBs 206 and 209), were inversely correlated with cellular ATP concentration (Table 3). There were no significant correlations between ATP and OCDD, BDE47, BPA, OCP or PFASs (Supplementary Fig. 1). The addition of “current smoker” as an adjustment to the model only marginally altered the results. When the TEQ values were grouped into TEQ_ortho_ and TEQ_planar_, only TEQ_planar_ was significantly correlated with ATP. As for the AhR bioactivity data, further adjustment for a history of myocardial infarction, stroke, heart failure, diabetes and medication for hypertension, diabetes or hyperlipidaemia only had a marginal effect on the correlations between ATP concentration and TEQ or individual POPs.

**Table 3:** Relationship of ATP concentration with TEQ or plasma levels of individual POPs (ln-TEQ, ln-POPs, pg/mL) are represented by regression coefficients (β) with corresponding 95% CIs and *P* values in linear regression models adjusted for sex, serum cholesterol, and serum triglycerides (n = 911).

Variable	β coefficient (95% CI)	*P* value
TEQ_total_	−1.84 (−2.71, −0.97)	0.000040*
TEQ_planar_	−1.80 (−2.65, −0.94)	0.000042*
TEQ_ortho_	−1.15 (−2.67, 0.37)	0.14
PCB74	−0.53 (−1.64, 0.59)	0.36
PCB 99	−0.055 (−1.035, .093)	0.91
PCB138	−.0167 (−1.44, 1.10)	0.80
PCB153	−0.73 (−2.14, 0.68)	0.31
PCB170	−1.15 (−2.74, 0.45)	0.16
PCB180	−0.98 (−2.55, 0.59)	0.22
PCB194	−0.27 (−0.99, 0.46)	0.47
PCB206	−1.51 (−2.97, −0.055)	0.042*
PCB209	−1.99 (−3.28, −0.70)	0.0025*
PCB105	−.023 (−1.18, 0.72)	0.63
PCB118	−0.33 (−1.40, 0.74)	0.55
PCB156	−0.98 (−2.41, 0.45)	0.18
PCB157	−0.71 (−2.05, 0.63)	0.30
PCB189	−0.66 (−1.51, 0.20)	0.13
PCB126	−1.22 (−1.84, −0.61)	0.00011*
PCB169	−1.857 (−3.08, −0.63)	0.0030*
OCDD	−0.48 (−1.41, 0.45)	0.31
BDE47	0.099 (−0.72, 0.92)	0.81

^*^Indicates significant *P* value. All variables were natural log-transformed.

CI, confidence interval; TEQ, toxic equivalence; PCB, polychlorinated biphenyl; TEQ_planar_: TEQ for dioxin-like coplanar, non-ortho-substituted PCBs; TEQortho: TEQ for dioxin-like mono-ortho-substituted PCBs; OCDD, octachlorinated dibenzo-p-dioxin; BDE, brominated diphenyl ether.

### TEQ did not have non-linear effects on AhR bioactivity or ATP concentration

**Figure 1:**
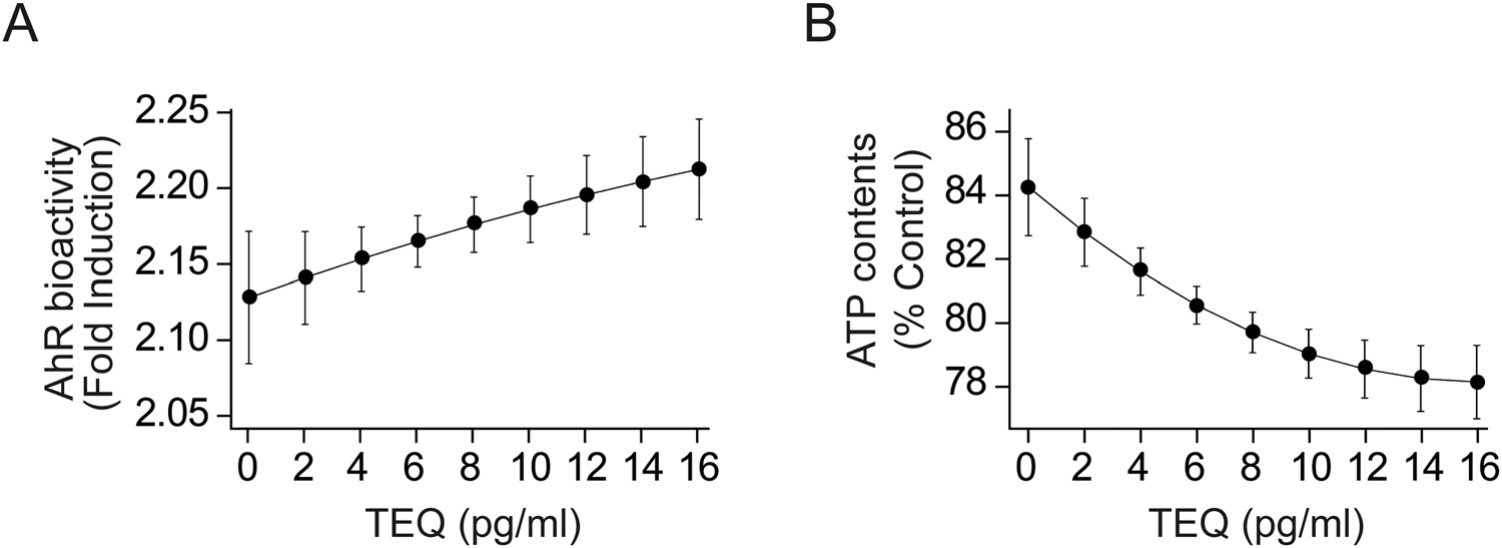
Relationship of AhR bioactivity or ATP concentration with TEQ_total_. (**A**) Relationship between TEQ_total_ and serum-induced AhR bioactivity which was measured by CALA assay. AhR bioactivity is presented as a fold induction over the AhR bioactivity of the CS-HS-treated control cells. (**B**) Relationships between TEQ_total_ and serum-induced ATP concentration. ATP content is presented as % Control of ATP content in control cells treated with 10% CS-HS. The predicted margins (and 95% confidence intervals) for AhR bioactivity or ATP concentration in serum-treated cells were calculated for given values of TEQ_total_ (pg/ml). The regression model used included a quadratic term for TEQ to account for curvilinear relationships. The model was adjusted for sex, serum cholesterol and serum triglycerides.

The relationships between TEQ_total_ and AhR bioactivity or ATP level were evaluated using linear regression models and the square of TEQ_total_ to search for non-linear effects. The equation used for these regression models was y = k_1_x + k_2_x^2^ + e, where y is AhR bioactivity or ATP level, k_1_ and k_2_ are regression coefficients, x is TEQ_total_ and e is a constant. The graphs in Fig. 1 show the expected values and 95% confidence intervals for AhR bioactivity (Fig. 1A) and ATP concentration (Fig. 1B) versus TEQ_total_ as regression lines. Both AhR bioactivity and ATP concentration had curvilinear relationships with TEQ_total_, but no non-linear effects were demonstrated. These data show that there was no asymmetric amplification or depletion of AhR bioactivity or ATP concentration at higher TEQ.

### Effects of four PCBs on AhR bioactivity and ATP concentration

**Figure 2:**
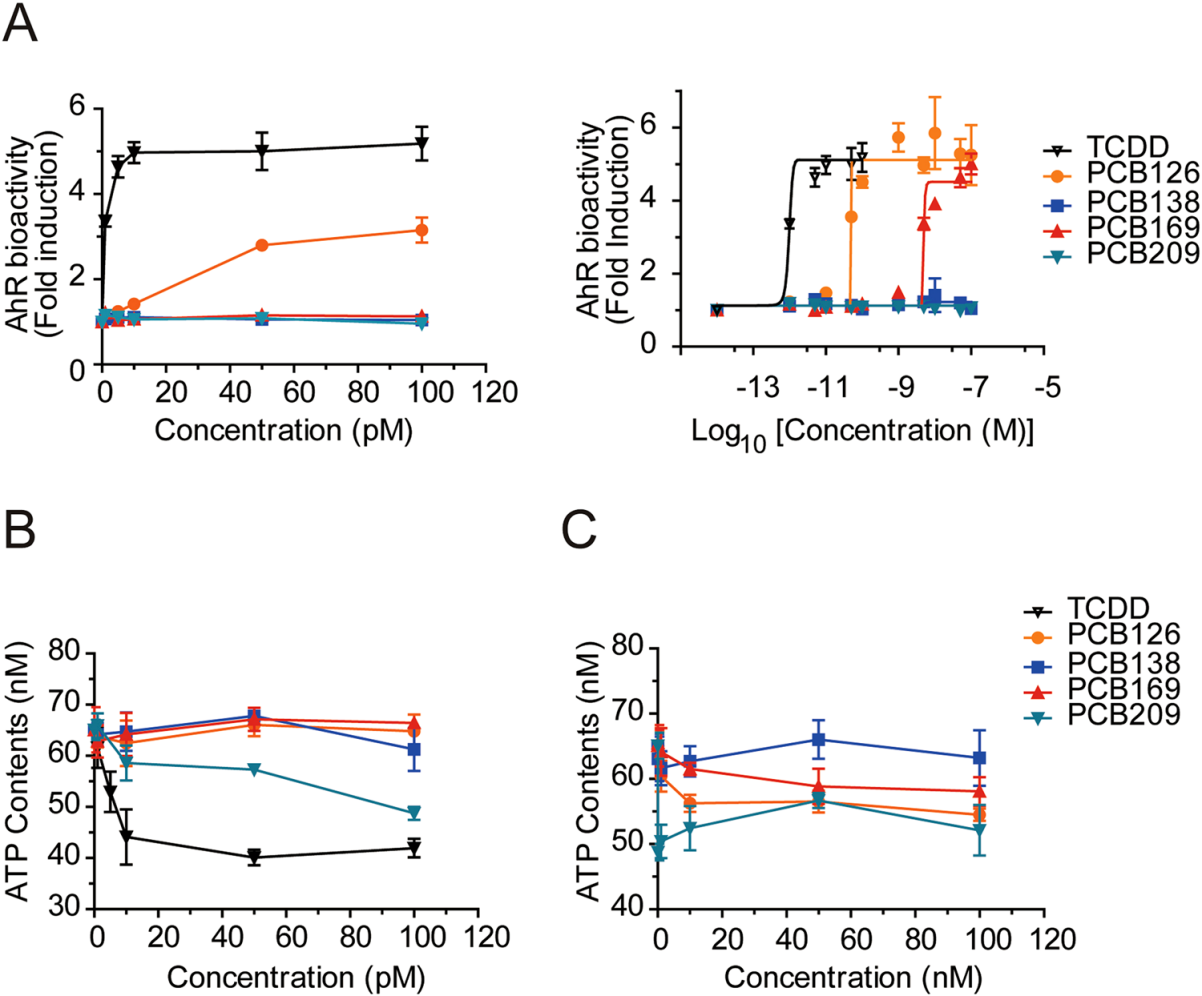
Dose-response curves for TCDD and four PCBs (126, 138, 169 and 209). (**A**) AhR bioactivity (left, pM ranges; right, log_10_ concentrations covering pM to nM ranges). AhR bioactivity is presented as a fold induction over the AhR bioactivity of the CS-HS-treated control cells. (**B**,**C**) ATP content (B, pM ranges; C, nM ranges). The intracellular ATP content of control cells was 65.1 ± 2.7 nM. The ATP concentration of 10% sample serum-treated cells was calculated as % Control of ATP content in control cells treated with 10% CS-HS and presented as nM which was calculated from the standard curve of ATP concentration (nM) = (% Control + 18.24) / 1.817 (Supplementary Fig. 2B,C). Only DLP (126, 169) activated AhR bioactivity, but both DLP (126, 169) and non-DLP (209) reduced ATP content. TCDD is a positive control. Data are mean ±SD (n = 6).

Of the PCBs detected in this study, we chose to analyse the effects of four PCBs (126, 138, 169 and 209) on AhR bioactivity and ATP concentration. PCB126 stimulated AhR-dependent bioactivity when present at >10 pM (Fig. 2A). The median plasma concentration of PCB126 in the PIVUS cohort was 40.4 pg/ml (123.8 pM) (Table 1), which was enough to activate AhR. This might explain why the PCB126 concentration in serum was significantly correlated with the serum-induced AhR bioactivity (Table 2). Conversely, the median plasma concentrations of other DLPs in the PIVUS cohort were lower than the required level to stimulate AhR bioactivity (Table 1). Indeed, PCB169 stimulated AhR at >10 nM (Fig. 2A), but its median plasma concentration in the PIVUS cohort was 171.4 pg/ml (475.0 pM). Non-DLPs (PCBs 138 and 209) did not activate AhR, although PCB138 was significantly correlated with AhR bioactivity (Table 2).

By contrast, cellular ATP concentration was significantly suppressed by three PCB congeners (126, 169 and 209) but not PCB138 at nM ranges (Fig. 2B,C, *P* < 0.01 vs. non-treated). The plasma concentrations of PCBs 126 and 169 were enough to reduce ATP concentration by 20%. Although the non-DLPs PCBs 206 and 209 were significantly associated with ATP concentration, their contribution to the suppression of ATP might be minimal because their median concentrations were as low as 57.7 and 52.5 pM, respectively. It should be noted that PCB138, which was significantly correlated with AhR bioactivity (Table 2), did not affect cellular ATP.

## Discussion

In the present study, we analysed the effect of serum from a large number of PIVUS cohort study samples on AhR bioactivity using CALA assays, and correlated these effects with the presence of various environmental pollutants. The results illustrate, for the first time, a positive linear relationship between CALA-determined AhR bioactivity and HRGC/HRMS-determined TEQ values, and reveal the contribution of specific PCBs to this AhR bioactivity. We conclude that serum-induced AhR bioactivity could be used as a reliable surrogate biomarker of the mixture of POPs in the circulation, thus reflecting their potential influence on human health. Because the CALA assay is inexpensive and requires only small amounts of serum, the technique could be applicable to both epidemiological studies and clinical settings.

Another important observation was that the ATP content of cells incubated with sample sera was inversely correlated with the TEQs derived from concentrations of the chemicals in the plasma. Thus, the toxicity of contaminated serum may involve the suppression of intracellular ATP production in target cells by POPs. Because intracellular ATP content is a good indicator of mitochondrial function^10^, we infer that serum POPs quantitatively inhibit mitochondrial function in the target cells. The effects of POPs on mitochondrial activity may occur through AhR-dependent and/or AhR-independent pathways, but specific involvement of the AhR in the effects of POPs on mitochondrial function remains to be studied in more detail.

Many individual POPs showed significant correlations with AhR bioactivity (Table 2). The interpretation of these correlations is complex because AhR bioactivity results from the cumulative effect of all the AhR ligands present, reflecting both the measured and unmeasured serum POPs. Six DLPs (PCBs 105, 118, 126, 156, 157 and 189) and six non-DLPs (PCBs 74, 99, 138, 153, 170 and 180) were significantly correlated with serum AhR bioactivity (Table 2). The plasma concentration of PCB126 (3,3′,4,4′,5-pentachlorobiphenyl, TEF = 0.1), the most potent DLP, was present within the range of concentrations required to activate AhR in a dose-dependent manner: 66.2 pM (21.6 pg/ml) in the 25% quartile; median, 123.8 pM (40.4 pg/ml); 220.6 pM (72.0 pg/ml) in the 75% quartile (Table 1). However, other DLPs, including PCB169 (3,3′,4,4′,5,5′-hexachlorobiphenyl, the second most potent DLP, TEF = 0.03), were found at sub-nM concentrations, at which they were not able to activate the AhR. Nevertheless, it is possible that the five DLPs could activate AhR in an additive or synergistic fashion, generating the positive correlation.

The significant associations between AhR bioactivity and non-DLPs are more difficult to understand than those with DLPs because non-DLPs do not activate AhR-DRE-dependent transcription. Because several reports show that non-DLPs interact with other nuclear receptors, such as oestrogen receptor, androgen receptor and the plasma thyroxine transport protein transthyretin, in *in vitro* profiling^18^, we checked whether non-DLPs might activate AhR-DRE-luc activity in the CALA assay, as DLPs did. Among the four tested PCB congeners (PCBs 126, 138, 169 and 209), only DLPs (PCBs 126 and 169) activated AhR-DRE-dependent transcription activity, whereas non-DLPs (PCBs 138 and 209) did not (Fig. 2). We conclude that only DLPs contributed to the CALA assay-determined serum AhR bioactivity and that PCB126 might be the principal compound responsible for the activation of AhR in the PIVUS cohort. Non-DLPs might simply be bystanders in these serum samples, although there were positive correlations between their concentrations and AhR bioactivity. In a cluster analysis of the various PCBs, we found that the less chlorinated PCBs form one cluster and the highly chlorinated PCBs another cluster^19^. It might be that within those two clusters some non-DLPs behave similarly to DLPs, explaining why at least some non-DLPs were correlated with AhR bioactivity in the present study.

Similar analyses of the relationships between ATP concentration and individual POPs demonstrated that ATP was negatively correlated with two coplanar DLPs, PCBs 126 and PCB169, and two non-DLPs, PCB206 (2,2′,3,3′,4,4′,5,5′,6-nonachlorobiphenyl) and PCB209 (decachlorobiphenyl) (Table 3). When DLPs were grouped according to the position of their chlorine substitution, only TEQ_planar_ (calculated from PCBs 126 and 169) was inversely correlated with ATP concentration, whereas both TEQ_planar_ and TEQ_ortho_ were significantly correlated with AhR bioactivity. In the cell-based ATP assay, the non-dioxin-like *ortho*-PCB138 (2,2′,3,4,4′,5′-hexachlorobiphenyl) did not suppress ATP production, but PCB209 did (Fig. 2). In order to validate the effects of PCBs on mitochondria, many different aspects of mitochondrial activities, such as mitochondrial membrane potential, oxygen consumption rate, oxidative phosphorylation, ATP generation, needed to be analyzed. Previously, we have shown that ATP contents and oxygen consumption rate are the best parameters reflecting mitochondrial activity^10^. When the oxygen consumption rates (OCR) of basal, ATP turnover, and maximum capacity of respiration, the physiological parameters of mitochondrial function, were measured using Seahorse XF-24 analyzer, DLP PCB126 and PCB169 as well as non-DLP PCB206 and PCB209 significantly reduced mitochondrial OCR at both low and high concentrations (Supplementary Fig. 1). Again, effects of PCB138 on OCR were marginal compared to other PCBs. The results support that the current assay on ATP contents is the valuable parameter to surrogate effects of serum on mitochondria.

Relationships between 18 POPs plus 12 environmental contaminants (BPA, three OCPs and eight PFASs) and AhR bioactivity or ATP concentrations were also analysed using the reported POP concentrations^16^. The chemical names and structures of all 30 compounds are shown in Supplementary Table 1. The *P*-values on a Log10 scale in a logistic regression model are shown as a Manhattan-type plot in Supplementary Fig. 2. Notably, PCB126 was significantly correlated with both AhR bioactivity and ATP concentration. Recently, it was reported that long-term exposure of rats to PCB126 induced obesity, impaired insulin sensitivity and induced oxidative stress in pancreatic β-cells^20^, while intraperitoneal exposure to PCB126 resulted in increased cardiovascular risk factors, heart weight, cholesterol, triglycerides and blood pressure^21^. Thus, the strong correlations between PCB126 and both AhR bioactivity and ATP concentration imply that exposure to PCB126 may be a major cause of mitochondrial dysfunction in the PIVUS cohort, leading to insulin resistance or cardiovascular defects.

OCDD, which was found in most subjects, was not significantly correlated with AhR activity. OCDD is the most prevalent dioxin in the environment, but its effect on AhR bioactivity is negligible, because its potency as a toxin is approximately 1,000–3,000 times weaker than that of TCDD^14^. BDE47 was positively correlated with AhR activation in the present study. BDE47 is the most abundant congener of polybrominated diphenyl ether (PBDE), but was shown not to induce AhR activation^22^ or CYP1A1 activity^23^. However, in another study in which the H4IIE-CALUX bioassay was used, BDE47 activated AhR as a full antagonist^24^. Thus, BDE47 may also be a relevant co-contaminant, because it has a similar contaminating pattern to the non-DLPs discussed above. Finally, neither OCDD nor BDE47 were correlated with ATP concentration, further indicating that AhR bioactivity may be connected to mitochondrial dysfunction. To draw conclusions regarding which POPs might be the culprits responsible for causing disease, direct cause-effect relationships between the individual POP and the disease of interest must be evaluated. Our findings suggest that mitochondrial inhibition, assessed by measuring ATP concentration, provides another useful toxicological assessment of the POP contamination of serum, independent of their activity as AhR ligands.

Polycyclic aromatic hydrocarbons (PAHs) are also AhR ligands. Although PAHs usually have a short half-life, it is unclear to what extent the short period of abstinence from smoking (overnight) applied in the present study played a role in the present study. However, only a small minority of the subjects in the present study were smokers.

In summary, the cell-based analysis of PIVUS cohort serum samples revealed that individual PCBs might affect serum AhR bioactivity and mitochondrial function differently. The combination of the two analyses suggested that PCBs 126 and 209 are major contributors to cellular AhR activation and ATP suppression, although statistically significant correlations were also observed between the concentrations of many POPs and these measures. In conclusion, we observed significant associations between TEQ and AhR bioactivity or ATP concentration, suggesting that these two cellular measurements may be valuable biomarkers for the toxic potential of serum POPs.

## Methods

### Study subjects

Participating subjects from the PIVUS cohort were aged 70 years (SD ± 1 month) and lived in Uppsala, Sweden, as described elsewhere in detail^15, 17, 25^. The subjects were randomly chosen from the study register, and 1,016 subjects participated (female, 50.2%), yielding a participation rate of 50.1%. This study was approved by the Ethics Committee of the University of Uppsala and the participants gave written informed consent.

All subjects were examined in the morning after an overnight fast. No medication or smoking was allowed after midnight. Participants were asked to complete a questionnaire regarding their medical history, smoking habits and regular medication. Lipid parameters (serum cholesterol and triglyceride) were measured using standard laboratory techniques^25^. Approximately 10% of the cohort reported a history of coronary heart disease, 4% reported stroke and 9% reported diabetes mellitus. Nearly half (45%) of the cohort reported the use of at least one medication for a cardiovascular condition, with anti-hypertensive medication being the most prevalent (32%). Fifteen per cent reported the use of statins, while insulin and oral antihyperglycaemic drug use was reported by 2% and 6% of the participants, respectively^26^.

### Chemicals

All chemical and assay kits were purchased from commercial sources. To measure AhR bioactivity and ATP concentration, four PCBs (126, 138, 169 and 209) and TCDD were purchased from ULTRA Scientific, N. Kingstown, RI, USA and Sigma-Aldrich, St. Louis, MO, USA, respectively. Each chemical was obtained in toluene, prepared in dimethyl sulfoxide (DMSO) at 10 μM (for TCDD) or 100 μM (for PCBs) and diluted in culture medium containing 10% CS-HS to the required concentrations. All experiments were performed in accordance with the relevant guidelines and regulations.

### Analysis of circulating POPs

Concentrations of circulating POPs were determined in stored plasma samples^15, 25, 27, 28^. Briefly, analysis was performed using solid-phase extraction and clean-up, followed by instrumental analysis on a HRGC/HRMS system (Waters, Milford, MA, USA), using a method slightly modified from that described^29^. The analytical method used to determine plasma concentrations of POPs in all samples was successfully validated with regard to recovery, accuracy and precision. Isotope dilution was used to quantify concentrations of 18 different POPs, including 16 PCB congeners (PCBs 74, 99, 126, 169, 118, 105, 153, 138, 156, 157, 180, 170, 189, 194, 206 and 209), OCDD and 2,2′,4,4′-tetrabromo diphenyl ether (BDE47) (a PBDE). The detection rate for all POPs was >95.5% except for OCDD (80.6%) and BDE47 (72.2%)^15^. Their concentrations are expressed as wet masses (pg/ml)^27^ and were adjusted for serum cholesterol and triglyceride in the statistical analysis. TEQ_total_ values were calculated using their respective TEFs, which express the toxicity of seven mono- and non-ortho-substituted DLPs (126, 169, 105, 118, 156, 157 and 189) and OCDD^30^. TEQs for the planar PCBs in this study (126 and 169) and ortho-PCBs in this study (105, 118, 156, 157 and 189) were calculated separately using the following formulae: TEQ_planar_ = (PCB126 × 0.1) + (PCB169 × 0.01); TEQ_ortho_ = (PCB105 × 0.0001) + (PCB118 × 0.0001) + (PCB156 × 0.0005) + (PCB157 × 0.0005) + (PCB189 × 0.0001); TEQ_total_ = TEQ_planar_ + TEQ_ortho_ + (OCDD × 0.001). Information on other environmental contaminants, including BPA, three OCPs and PFASs, was also included for comparison with PCBs^16, 17^.

### Plasmid preparations

The pGL4-DRE-luc(puro+) reporter plasmid was constructed in pGL4-basic(puro+) (Promega, Madison, WI, USA). The DRE fragment of murine CYP1A1 was amplified from pGL3-CYP1A1-luc^10^. The pRL-mTK *Renilla* reporter plasmid containing the minimal promoter of *Herpes simplex* virus thymidine kinase (minimal TK) was obtained from Dr Sehyung Cho’s laboratory (Kyung Hee University, Seoul, Korea)^31^.

### Serum preparation

Serum was prepared by allowing the blood to clot and then removing the clot by centrifugation. Aliquots (0.5 ml) of serum were stored at −70 °C until use. Freeze-thaw cycles were avoided. Frozen serum was quickly thawed at 37 °C, heat-inactivated at 56 °C for 30 min and centrifuged at 12,000 × *g* for 2 min. The cleaned supernatant serum was collected and confirmed to be free of bacterial contamination by incubation in culture media for 24 h.

Dextran-coated charcoal was prepared by stirring 0.5 g of charcoal overnight at 4 °C with 0.05 g of dextran T500 (Sigma-Aldrich, St. Louis, MO, USA) in 200 ml of 0.1 M Tris-HCl (pH 8.0)^32^. The suspension was centrifuged at 1,000 × *g* for 10 min, and the supernatant was discarded. The dextran-coated charcoal pellets were mixed with 50 ml of standard human serum, which was donated by a healthy man, and incubated at 45 °C for 45 min. Charcoal was then removed by centrifugation at 1,000 × *g* for 10 min, and the sera were sterilised by passing through 0.45 μm syringe filters. The CS-HS was then heat-inactivated at 56 °C for 30 min for use as control serum. Aliquots of this serum were stored at −70 °C until use.

### AhR bioactivity assay

Hepa1c1c7 mouse hepatoma cells (CRL-2026) were cultured in α-minimum essential medium supplemented with 10% foetal bovine serum and 1% penicillin/streptomycin at 37 °C in a 5% CO_2_ atmosphere. Cells seeded at 1×10^5^/well in a 6-well plate were co-transfected with pGL4-DRE-luc(puro+) and pRL-mTK using Superfect (Qiagen, Valencia, CA, USA)^10^. Puromycin (1 μg/ml)-resistant stable colonies were selected for 3 weeks, and then clones showing TCDD-dose-dependent activity were selected for further use.

The modified CALA assay^10^ was performed to quantify DRE-dependent luciferase activity. Briefly, the 70% confluent, stable cells were treated for 24 h with 10% heat-inactivated human serum samples (10 μl) or 10% CS-HS (control) in phenol red-free Dulbecco’s modified Eagle medium. Luciferase activities were measured using a Dual-Glo Luciferase assay system (Promega, Madison, WI, USA) and luminometer (Berthold, Bad Wildbad, Germany) and subsequently normalised against *Renilla* luciferase activity. AhR bioactivities of serum-treated cells are presented as a fold induction over the AhR bioactivity of the 10% CS-HS-treated control cells. AhR bioactivity was induced by 1.68 ± 0.34-fold by standard human serum prior to charcoal stripping. Standard curves were prepared using luciferase activities (AhR bioactivity) of the cells exposed to serially diluted TCDD (0–50 pM) for 24 h in the presence of 10% CS-HS (Supplementary Fig. 3A). When the AhR bioactivity in a 10% serum sample was converted to TCDD equivalents (TCDDeq, pM) using the standard curve (0–10 pM TCDD) (Supplementary Fig. 3A, inset), a 0.1-fold induction of AhR bioactivity was equivalent to 0.37 pM TCDDeq. Thus, for example, the difference between a 2.0-fold induction (3.283 pM TCDDeq) and a 2.1-fold induction (3.653 pM TCDDeq) is equivalent to 0.370 pM TCDDeq. All assays were performed in duplicate. Similarly, the dose-dependent AhR bioactivities of four PCBs (126, 138, 169 and 209) and TCDD were compared by incubating the cells for 24 h with various concentrations of each in the presence of 10% CS-HS.

### ATP content assay for the mitochondrial inhibition induced by serum samples

The mitochondrial inhibition induced by human serum samples was evaluated by determining intracellular ATP content, as described previously^10^. Briefly, Hepa1c1c7 cells were co-transfected with pRL-mTK and pcDNA3.1(neo + ) using Superfect (Qiagen, Valencia, CA, USA). G418-resistant stable colonies were selected for 3 weeks, and then the clones showing stable *Renilla* luciferase activity were selected. Stable clones (5×10^4^/well) were treated with 10% human serum samples or 10% CS-HS for 48 h in 96-well plates. The intracellular ATP content of the treated cells was determined using the luciferin-luciferase reaction and CellTiter-Glo luciferase kits (Promega, Madison, WI, USA)^33^. *Renilla* luciferase activity was measured by adding an equal amount of Stop & Glo substrate solution from the Dual-Glo Luciferase assay kit (Promega, Madison, WI, USA). ATP concentrations were normalised to *Renilla* luciferase activity. The dose-dependent effects of four PCBs (126, 138, 169 and 209) and TCDD on ATP concentration were also determined in the presence of 10% CS-HS. All data are presented as % Control of ATP content in control cells treated with 10% CS-HS. The intracellular ATP content of control cells was 65.1 ± 2.7 nM. The ATP concentration of 10% sample serum-treated cells was calculated from the standard curve of ATP concentration (nM) = (% Control + 18.24) / 1.817 (Supplementary Fig. 3B,C). The intra- and interassay coefficients of variation of these methods were less than 6.0%.

### Statistical analysis

All variables were evaluated for normality by Shapiro-Wilk’s W test, and variables with a skewed distribution were natural log-transformed^34^. These included all the POPs and calculated TEQs. Separate linear regression models with AhR bioactivity and ATP concentration as dependent variables were used to evaluate their relationships with log_e_-TEQs or concentrations of individual POPs as independent variables, adjusting for sex, serum cholesterol and serum triglycerides. The possibility of a sex interaction of AhR bioactivity with TEQ was investigated by including a specific interaction term in the regression model. Thereafter, the possibility of a non-linear relationship between TEQ and AhR bioactivity or ATP concentration was also evaluated by including a quadratic term for TEQ in the model.

The primary hypothesis tested in the current study was that the calculated TEQ was directly related to AhR bioactivity and ATP concentration. Thus, given the two outcomes tested, the critical *P-*value was set as 0.025, according to the Bonferroni correction for multiple testing. Evaluation of the individual POPs was regarded as supplementary information and was not subjected to any formal adjustment for multiple testing.

Statistical differences between experimental groups were assessed by Student’s *t*-tests using InStat (GraphPad Software, San Diego, CA, USA). Values of *P* < 0.05 were considered statistically significant.

## Electronic supplementary material

Supplemental Data
