# Supplementary material for: Relationships between serum-induced AhR bioactivity or mitochondrial inhibition and circulating polychlorinated biphenyls (PCBs)
Source: Sci Rep. 2017 Aug 24;7:9383. doi: 10.1038/s41598-017-09774-1 (PMC5571204; doi:10.1038/s41598-017-09774-1)
Supplement: Supplementary file 1 — Supplemental Data [file 41598_2017_9774_MOESM1_ESM.pdf]

Relationships between serum-induced AhR bioactivity or mitochondrial inhibition and circulating polychlorinated biphenyls (PCBs)

Wook Ha Park[1], Sora Kang[1], Hong Kyu Lee[2], Samira Salihovic[3], Bert van Bavel[3], P. Monica Lind[4], Youngmi Kim Pak[1*], Lars Lind[5*]

Supplementary Figures

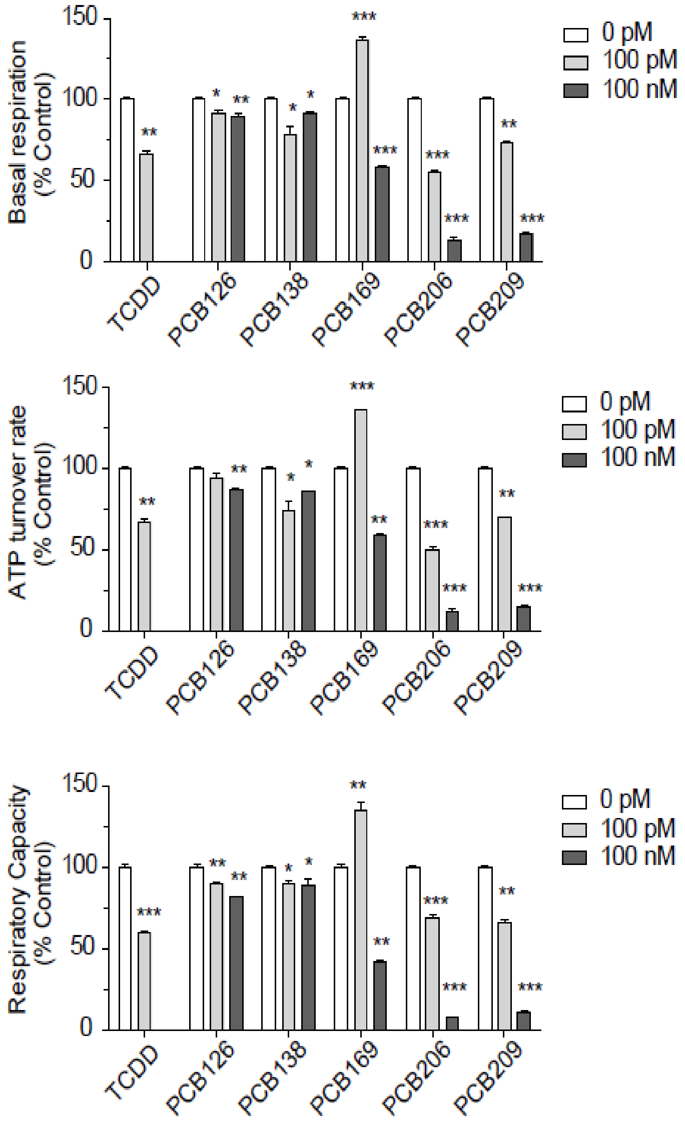

Supplementary Figure 1. Effects of TCDD and five PCBs (126, 138, 169, 206 and 209) on oxygen consumption rate

Hepa1c1c7 cells were treated with TCDD or PCBs (126, 138, 169, 206, or 209) at 100 pM or 100 nM for 24 h. Mitochondrial respiration reflected by oxygen consumption rates (OCR) level was determined using Seahorse XF-24 analyzer. Oligomycin (1 μM), the uncoupler FCCP (0.3 μM), and the electron transport inhibitor rotenone (1 μM) were consecutively injected to obtain ATP turnover rate and total respiratory capacity after measurement of basal respiration (basal OCR) as previously described[1]. *p<0.05, **p<0.01, ***p<0.001 vs. the DMSO-treated control, n=6

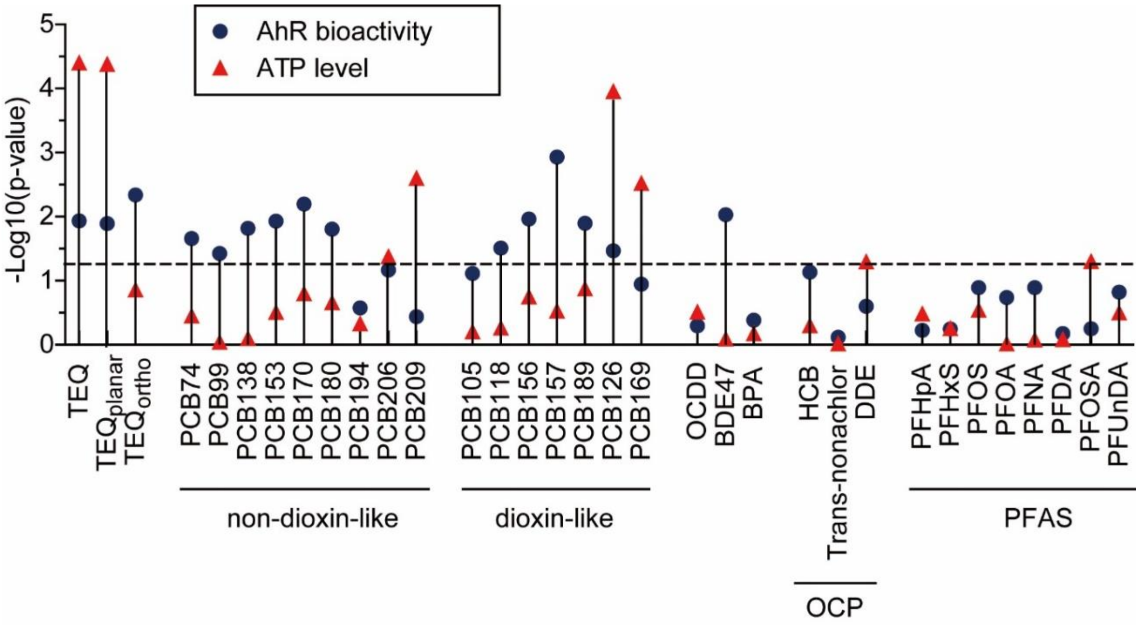

Supplementary Figure 2.

“Manhattan” type plot showing the p-values (expressed at the −log10 scale) in TEQs, 16 PCBs and 14 environmental contaminants vs. AhR bioactivity (circle) or ATP contents (triangle). Variables above the broken line denotes variables with $p<0.05$. BPA, bisphenol A; OCP, organochlorine pesticides; HCB, hexachlorobenzene; DDE, p,p′-DDE; PFAS, perfluoroalkyl substances; PFHpA, Perfluoroheptanoic acid; PFHxS, Perfluorohexane sulfonic acid; PFOS, Perfluorooctane sulfonic acid; PFOA, Perfluorooctanoic acid; PFNA, Perfluorononanoic acid; PFDA, Perfluorodecanoic acid; PFOSA, Perfluorooctane sulfonamide; PFUnDA, Perfluoroundecanoic acid. For abbreviations of other variables, see Table 1.

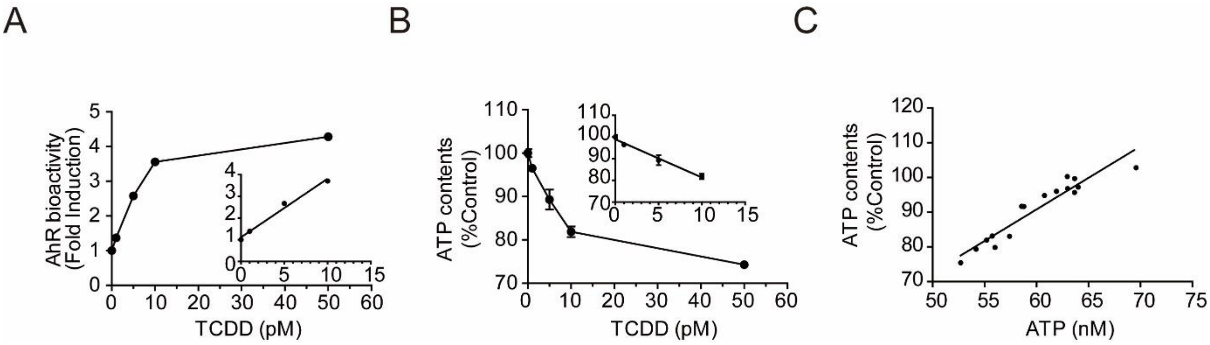

Supplementary Figure 3. Standard curves.

(A) Standard curve of AhR bioactivity by CALA assay in response to TCDD in the presence of 10% CS-HS. Inset graph is the linear range of AhR binding activity between 0 and 10 pM of TCDD. The line equation of the linear regression is y=0.2702x+1.113.

(B) Standard curve of ATP contents (% Control of DMSO-treated cells) by luciferin-luciferase reaction in response to TCDD in the presence of 10% CS-HS. Inset graph is the linear range of ATP contents (% Control) between 0 and 10 pM of TCDD.

(C) Standard curve to calculate ATP concentration from % Control of ATP contents obtained from graph B. ATP concentrations (nM) were calculated from luminescence of ATP standard solutions. % Control of ATP contents was calculated from luminescence of DMSO control cells normalised by renilla activities.

Supplementary Table 1. Chemical names and structures of 30 POP compounds analyzed in the study.

POPs	Chemical names	Chemical strucures
PCB74	2,4,4',5-Tetrachlorobiphenyl	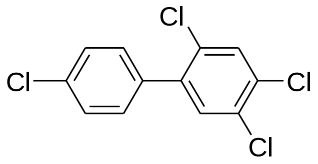
PCB99	2,2',4,4',5-Pentachlorobiphenyl	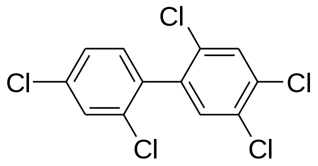
PCB138	2,2',3',4,4',5-Hexachlorobiphenyl	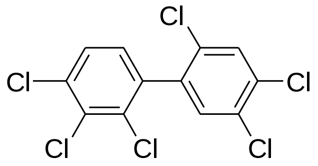
PCB153	2,2',4,4',5,5'-Hexachlorobiphenyl	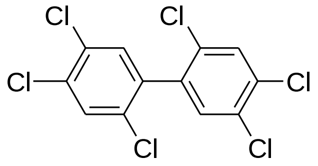
PCB170	2,2',3,3',4,4',5-Heptachlorobiphenyl	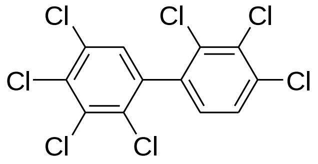
PCB180	2,2',3,4,4',5,5'-Heptachlorobiphenyl	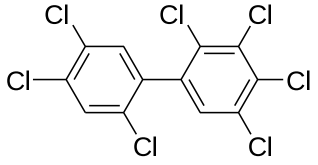
PCB194 [a]	2,2',3,3',4,4',5,5'-Octachlorobiphenyl	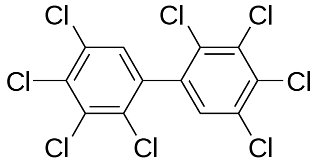
PCB206	2,2',3,3',4,4',5,5′,6-Nonachlorobiphenyl	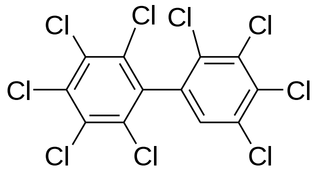

PCB209	Decachlorobiphenyl	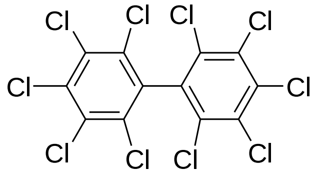
PCB105	2,3,3',4,4'-Pentachlorobiphenyl	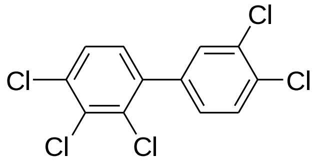
PCB118	2,3‘,4,4‘,5-Pentachlorobiphenyl	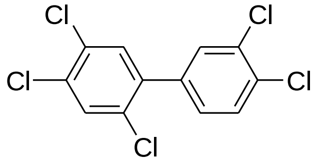
PCB156	2,3,3',4,4‘,5-Hexachlorobiphenyl	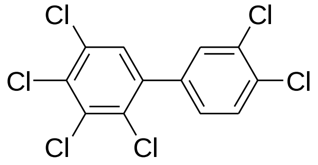
PCB157	2,3,3',4,4‘,5'-Hexachlorobiphenyl	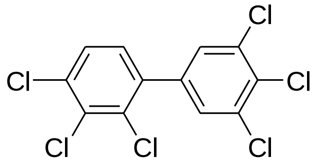
PCB189	2,3,3',4,4‘,5,5'-Heptachlorobiphenyl	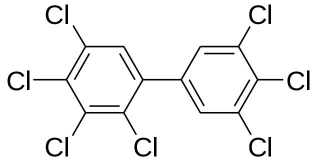
PCB126	3,3',4,4‘,5-Pentachlorobiphenyl	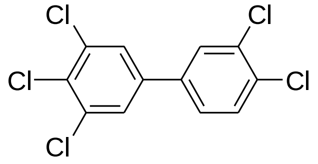
PCB169	3,3',4,4‘,5,5‘-Hexachlorobiphenyl	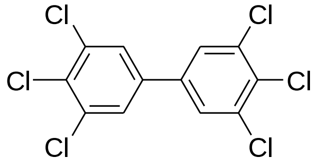

OCDD	Octachlorodibenzo-p-dioxin	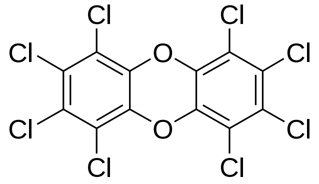
BDE47	2,2´,4,4´-tetrabromodiphenyl ether	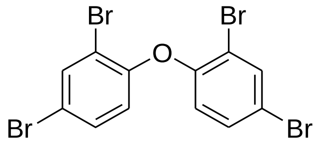
BPA	Bisphenol A	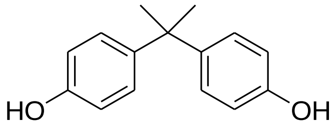
HCB	Hexachlorobenzen	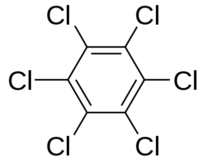
TNC	Transnonachlor	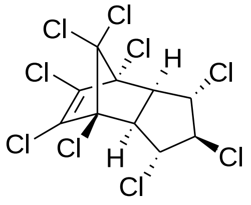
DDE	Dichlorodiphenyldichloroethylene	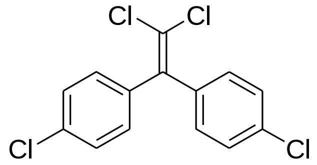
PFHpA	Perfluoroheptanoic acid	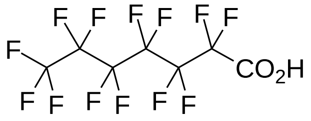
PFHxS	Perfluorohexane sulfonate	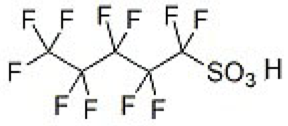
PFOS	Perfluorooctanesulfonic acid	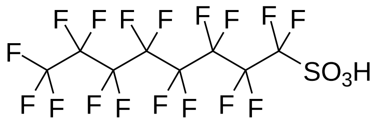

PFOA	Perfluorooctanoic acid	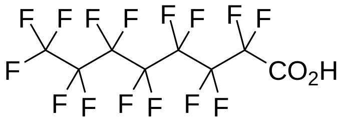
PFNA	Perfluorononanoic acid	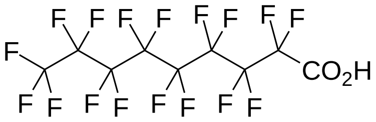
PFDA	Perfluorodecanoic acid	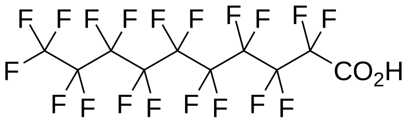
PFOSA	Perfluorooctanesulfonamide	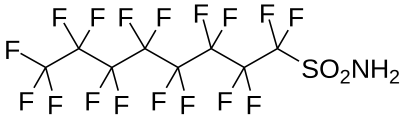
PFUnDA	perfluoroundecanoic acid	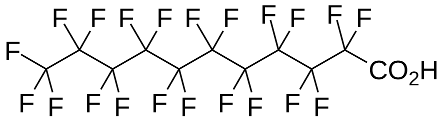

Supplementary Method

Measurement of oxygen consumption rate using Seahorse XF-24 analyzer

Oxygen consumption rate (OCR, pmoles/min) was measured using Seahorse XF-24 analyzer (Seahorse Bioscience, Billerica, MA). Hepa1c1c7 cells (4×10^3/well) were seeded in XF-24 microplates in 250 μl DMEM containing 10% designated human sera or TCDD in 10% CS-hS and incubated at 37°C/5% CO_2 for 24 h. Assays were initiated by removing the media from each well and replacing it with 675 μl of assay media (DMEM without sodium bicarbonate) pre-warmed to 37°C. Following gentle mixing for 10 min in XF-24 Analyzer, the baselines of OCR (basal OCR) were measured simultaneously for 3 min. Inhibitors were consecutively injected into each well to reach the desired final working concentrations (1 μg/ml oligomycin, 0.3 μM carbonylcyanide-p-trifluoromethoxy phenylhydrazone (FCCP), and 0.1 μM rotenone). ATP turnover rate and total respiratory capacity were calculated by subtracting oligomycine-OCR from basal OCR, and rotenone-OCR from FCCP-OCR, respectively[2]. Results were normalized by cell numbers. Measurements were performed in duplicate (n=3) and all data were presented as % Control.

References

1 Park, W. H. *et al.* Novel cell-based assay reveals associations of circulating serum AhR-ligands with metabolic syndrome and mitochondrial dysfunction. *Biofactors* **39**, 494-504, doi:10.1002/biof.1092 (2013).

2 Birket, M. J. *et al.* A reduction in ATP demand and mitochondrial activity with neural differentiation of human embryonic stem cells. *J Cell Sci* **124**, 348-358, doi:124/3/348 [pii] 10.1242/jcs.072272 (2011).
